# Virological Remission in Two HIV-Infected Children After Early cART in Libreville, Gabon

**DOI:** 10.1155/crpe/5554276

**Published:** 2025-09-26

**Authors:** B. Bivigou-Mboumba, C. Eyi Zang, P. Moussavou-Boundzanga, Y. Loumouamou, J. F. Djoba Siawaya, S. Ategbo

**Affiliations:** ^1^Joint Research Unit Between the Interdisciplinary Center for Medical Research of Franceville and the Military Health Service, Libreville, Gabon; ^2^Paediatric Service, Mother-Child University Hospital Center of Jeanne EBORI Foundation, Libreville, Gabon

**Keywords:** HIV, HIV DNA PCR, infant, seroreversion, virological remission

## Abstract

**Introduction:** Early antiretroviral therapy has a positive impact on the follow-up of an HIV-positive child, allowing assessment of the child's clinical and biological course. We report two cases to highlight the effects of early triple antiretroviral therapy (cART) on viral remission with seroreversion of HIV-infected infants in Gabon.

**Case Presentation:** We present two cases of infants born to HIV-infected mothers in Libreville (Gabon). The first infant was referred to the “Center Hospitalier Universitaire Mère-Enfant” (CHUME FJE) with a positive PCR result. He was born to a mother living with HIV, whose adherence to ART had been intermittent (7th and 9th months of pregnancy). Delivery was vaginal. At birth, nevirapine was administered discontinuously for 6 weeks. After a positive GenXpert PCR at 7 weeks, triple therapy was started with abacavir–lamivudine and ritonavir-boosted lopinavir (2IN-1IP). At 2 months, he was asymptomatic, and his clinical and laboratory parameters were normal. A second PCR at another Level 3 reference laboratory (CIRMF) confirmed HIV infection. We switched the antiprotease with an anti-integrase (dolutegravir, which was available). After 9 months of treatment, the patient's nutritional status was considered satisfactory, and the DNA PCR performed on GenXpert was negative. The second infant born to a mother living with HIV was admitted for posthospital monitoring of perinatal asphyxia. He was born by caesarean section, and nevirapine had been administered from birth. He was put on ART after two positive PCRs with zidovudine–lamivudine –nevirapine. At 4 months, the GenXpert DNA PCR became negative.

**Conclusion:** Virological remission with seroreversion of a previously HIV-infected infant is possible in Gabon. Further immunological (Ac assay) and virological (ultrasensitive proviral DNA on blood mononuclear cells) tests are needed in this infant to determine his definitive status.

## 1. Background

In 2021, 1.7 million children aged between 0 and 14 were infected with HIV [1]. HIV remains a public health problem worldwide. West and Central Africa account for 8% of the world's population and 22% of deaths [1]. Mother-to-child transmission (MTCT) remains predominant in our context [2–4].

The benefits of immediate and early ART are to halt the clinical progression of the infection, reduce the viral reservoir in infants, and restore CD4 T lymphocytes more rapidly [5–7].

The latent reservoir is a real obstacle to achieving remission from this scourge [8–10]. Plasmatic HIV-1 RNA is the biomarker that determines viral suppression, and HIV-1 DNA is the biomarker used to estimate the size of the reservoir [11].

Seroreversion is defined as negative serology in an infected person, and a few rare cases have been described worldwide, including in the USA, Canada, and Australia [10, 12].

In these cases, we emphasize the virological remission observed in infants born to HIV-infected mothers. These infants, initially diagnosed as positive through PCR testing, presented after undergoing a 9-month and a 4-month antiretroviral therapy (ART) regimen. They subsequently received a negative serological test, which was conducted twice at the Center Hospitalier Universitaire Mère-Enfant (CHUME FJE) in Libreville.

## 2. Cases Presentation

The 2-month-old infant was referred from the Laboratoire National de Santé Publique (level 3 laboratory) to the CHUME FJE with a positive PCR result. He was born to a 34-year-old HIV-positive mother who had been on dolutegravir–tenofovir–lamivudine for 6 years. She had a break in combined antiretroviral therapy (cART) during two months (from the 7th to the 9th month) of pregnancy, during the COVID-19 pandemic. This cART was consecutive to the stock-outs. The viral load at 33 weeks' gestation was not taken.

The baby was born at term by vaginal delivery with a weight of 3240 g. Nevirapine prophylaxis was given from birth but administered discontinuously for 6 weeks. At 7 weeks of age, cART was started with abacavir–lamivudine–lopinavir boosted with ritonavir after a 1st positive PCR (GenXpert DNA PCR). A 2nd control PCR was carried out at 3 months of age, confirming HIV infection. The antiprotease was switched to dolutegravir, which was available at the time. Hematology and biochemistry were both normal. At 4 and 9 months, the CD4 count was low (299–342 mm^3^) and the CD4/CD8 ratio increased from 0.46 to 0.55. At 11 and 12 months, the two qualitative PCRs performed on the GeneXpert HIV-1 Qual test (LD: 203 cp/mL) were negative, and the Determine RDT (Abbott) performed was also negative. At 12 months, the plasma viral load (PVL) and cellular viral load (CVL) were undetectable (LD: < 40 cp/mL). In the same month, nested PCR with an accurate detection, targeting the polymerase and envelope, was also undetectable. We concluded that viral remission with seroreversion had occurred.

The second infant was viewed as a 20-day-old newborn, brought to a posthospitalization consultation for control of perinatal asphyxia in an HIV-infected newborn (2 PCR positive), born to a known seropositive mother who was being monitored. He was born at term by cesarean section, with poor adaptation to extrauterine life and a birth weight of 2600 g. The indication for cesarean section was a placenta previa, associated with large myomas and a transverse position of the fetus. In addition, the viral load was not taken at the end of the pregnancy. The mother was 37 years old and had been seropositive for 14 years. Administration of cART was discontinued, with a resumption in 2020 of dolutegravir-tenofovir-lamivudine. She also underwent a shortage of cART during 2 months (from the 6th to the 8th month of pregnancy). The newborn had received NVP prophylaxis since birth. Early screening was carried out during hospitalization on Days 6 and 9 of life and was positive. He was hospitalized for 17 days. He was brought to the clinic at 20 days of age for a posthospital check-up as part of the follow-up of perinatal asphyxia in an HIV-infected infant. The patient was still on antiretroviral prophylaxis. He started cART at 2 months of age, 6 weeks after diagnosis, with zidovudine–lamivudine–nevirapine. The reasons for the delay in starting cART were the difficulty in finding a formulation suitable for his weight (*p* = 2.5 kg) and the mother's failure to keep appointments. From 20 days to 5 months of age, he showed psychomotor retardation and moderate malnutrition. Qualitative PCR performed on GeneXpert HIV-1 Qual test (LD: 203 cp/mL) at 4 and 5 months of age was negative on two occasions.

At 5 months, CVP and CVC were both undetectable (LD: < 40 cp/mL), and the determination was negative. A nested PCR targeting polymerase and envelope performed at 5 months was negative.

## 3. Discussion

### 3.1. MTCT and Risk Factors

Both mothers experienced a two-month shortage of cART due to a national cART supply disruption, a negative consequence of the COVID-19 pandemic. The mother of the first case of virological remission with seroreversion described in 2013, the Mississippi case, had not received ART before or during pregnancy [3, 10, 13, 14].

At birth, HIV-1 infection occurs in an immunologically naive environment, where maternal ART and immune responses during pregnancy significantly influence the formation of the viral reservoir [15].

The discontinuity of cART has contributed to this transmission. Chibwesha et al. [16] showed that the duration of antenatal cART of at least 13 weeks prevented MTCT. The other risk factors found were the shortage of ART, which remains to be resolved, and unknown maternal viremia at the end of pregnancy. High maternal PVL (PLVL) remains the main biological predictor of MTCT as described by Hoffman et al. in 2010.

Noncompliance with ART, neonatal resuscitation, and exclusive artificial feeding argue for perinatal rather than postnatal MTCT [3, 13]. Prophylaxis was used for both infants to prevent the transmission of the virus.

### 3.2. Diagnosis and Early cART

These two cases meet the standard diagnostic criteria for HIV-1 infection established by the Program National de Lutte contre les STI et le VIH/SIDA (PNLIST, Gabon) [17].

Case 1 was initiated with 2 nucleoside reverse transcriptase inhibitors (NRTIs) combined with a protease inhibitor (PI) and subsequently switched to an integrase inhibitor (II) due to its availability at the time. The 2nd was put on 3INTI because of his low weight and the unavailability of formulations suitable for a weight of 2.5 kg. Cases from Mississippi, Canada, and London were also treated with nucleoside inhibitors and antiprotease with the same efficacy but with different delays [18].

The 1st infant had a low CD4 T count with no clinical manifestations. These figures are in partial agreement with the trend observed by Pierre Frangé et al., who found that in children, early ART was not significantly associated with higher CD4 levels.

Early initiation of cART at the onset of HIV infection may limit the establishment of the HIV reservoir and improve clinical outcomes [19, 20], as was the case in our 2 patients.

### 3.3. Early cART and Viral Reservoir

The HIV-1 reservoir seems to be limited for infants; this means that when early cART with long-term viral suppression is started in these children, the size of the HIV reservoir is smaller and less diverse than in adults [5, 7]. This illustrates the interaction between early virological remission and the size of the viral reservoir. There is a concordance between the size of the latent reservoir and the time to the first undetectable viral load.

### 3.4. Early cART and Viremia

Undetectable viremia was observed after 9 months of cART for the first case and after 2 months for the second. The same finding was made in the Mississippi child but earlier (1 month after cART), at 6 months for the Canadian case and at 3 months for the Milan case [5, 21]. The Mississippi case had received 30 h of cART at the 18th month of life, and the other 2 cases before the 24th hour of life until the age of 3 years. In all cases, viral suppression was effective within 6 months of ART.

cART disintegrated the proviral DNA. When cART is initiated rapidly, HIV-1 DNA disintegrates rapidly. One hypothesis is the death of productively infected cells, the loss of unintegrated virus, and the simultaneous blockage of newly infected cells [12, 21].

### 3.5. Early cART and Seroreversion

The 2 cases described showed seroreversion, as did children in Mississippi, Milan, Canada, South Africa, and France [7, 18].

Suppression after perinatal HIV infection results in negligible peripheral blood proviral reservoirs in adolescence and is associated with negative or indeterminate serostatus. These results are encouraging and highlight the long-term effect of effective early control of HIV replication and the usefulness of HIV serostatus as a biomarker for small proviral reservoir size, but not necessarily for cure. The absence of HIV-specific antibodies in this context probably reflects early control of virus replication with limited exposure to HIV antigens [22].

### 3.6. Limitations

We had only two cases out of the 5 infected children who had benefited from early screening. The mothers' CD4 cell counts and viral loads had not been carried out. Samples of the initial positive tests were not kept. Immunological monitoring of the children was incomplete due to a lack of financial resources. Clinicobiological tracking remains to be carried out before complete remission can be said to have been achieved.

## 4. Conclusion

The two cases reported here are the first documented cases in Gabon, adding to the so-called “functional” remissions range. Virological remission and seroreversion of an infant previously infected with HIV are possible and accessible to all, even in countries with limited resources. This remission is possible, provided screening is coupled with early and effective management of identified cases. However, we must remain cautious because of the current global data and continue immunological and molecular investigations to determine the definitive status of the two infants. Then, we need ultrasensitive proviral DNA assays and formal ELISA or western blot serological testing to confirm the loss of antibodies and to continue monitoring for any viral rebound.

## Data Availability

Data are available upon request.
